# Development of Mesopore Structure of Mixed Metal Oxide through Albumin-Templated Coprecipitation and Reconstruction of Layered Double Hydroxide

**DOI:** 10.3390/nano11030620

**Published:** 2021-03-02

**Authors:** Sang-Yong Jung, Bo-Kyung Kim, Hyoung-Jun Kim, Jae-Min Oh

**Affiliations:** 1 Department of Energy and Materials Engineering, Dongguk University-Seoul, Seoul 04620, Korea; syjung05@naver.com; 2 JOIN International Ltd. (JIL), Samsung Cheil Bldg., 18th Fl. 309, Teheran-ro, Gangnam-gu, Seoul 06151, Korea; bkkim_@naver.com; 3 Research Institute, National Cancer Center, 323 Ilsan-ro, Goyang, Gyeonggi 10408, Korea

**Keywords:** mixed metal oxide, layered double hydroxide, albumin, sacrificial template, porous structure

## Abstract

Mixed metal oxide (MMO) with relatively homogeneous mesopores was successfully obtained by calcination and reconstruction of albumin-templated layered double hydroxide (LDH). The aggregation degree of albumin-template was controlled by adjusting two different synthesis routes, coprecipitation and reconstruction. X-ray diffraction patterns and scanning electron microscopic images indicated that crystal growth of LDH was fairly limited during albumin-templated coprecipitation due to the aggregation. On the hand, crystal growth along the lateral direction was facilitated in albumin-templated reconstruction due to the homogeneous distribution of proteins moiety. Different state of albumin during LDH synthesis influenced the local disorder and porous structure of calcination product, MMO. The N_2_ adsorption-desorption isotherms demonstrated that calcination on reconstructed LDH produced MMO with large specific surface area and narrow distribution of mesopores compared with calcination of coprecipitated LDH.

## 1. Introduction

Mixed metal oxide (MMO) is one of the widely utilized inorganic materials as catalyst, electrode, adsorbent, etc. [1,2,3]. The MMOs containing transition metals such as Fe, Ni, Mn, and Co, due to the various oxidation state and gas adsorption property of those metal species, have been reported as catalysts for oxidation reaction [4], methane decomposition [5], and hydrogenation [6]. The MMOs consisting of main group elements like Mg and Al were often reported as acid catalysts [7] or adsorbents for graphene oxide [8], atmospheric CO_2_ [9], and dye [10]. Taking into account the aforementioned application of MMOs, it is important to not only control metal composition but also design high specific surface area with controlled porosity. To address the demand, there have been various synthesis methods developed to produce MMOs with intended properties. The ceramic method, in which two or more metal oxide components are mixed and thermally treated, is the most common method [11,12]; however, it takes high energy to facilitate diffusion of metal species between different lattice structures. The sol-gel process, which consists of conversion of monomer to colloid (sol) and further aging to integrated network (gel), is another method to prepare MMO [13,14,15].

Recently, calcination of layered double hydroxide (LDH) is suggested as an alternative way to produce MMO. The LDH is composed of positively charged mixed metal hydroxide layers and charge compensating interlayer anions [16,17]. A layer of LDH is expressed as the chemical formula of M(II)_1−x_M(III)_x_(OH)_2_^x+^ where the x value is variable within the range 0.2–0.4 [18]. The M(OH)_6_ octahedrons are connected by edge-sharing manner throughout the crystallographic *ab*-plane direction, giving rise to thin layer with ~0.5 nm thickness and hundreds of nanometers lateral size. According to previous literature, calcination of LDH takes place through the following steps. At low temperature (<200 °C), dehydration of surface and interlayer water occurs; at moderate temperature (200–600 °C), dehydroxylation and gasification of interlayer anion occurs simultaneously, giving rise to the phase transformation to MMO [19,20]. This phase transformation accompanies slight collapse of layered structure, which is mediated by the evolution of small metal oxide domains and partial migration of M(III) from octahedral to tetrahedral site [21,22]. As the layered structure could be partly preserved after calcination, the MMO obtained from LDH precursor is often referred to as layered double oxide (LDO). Calcination of LDH is advantageous in the production of MMO due to the following reasons. First, the LDO material contains homogeneous distribution of metal components as both divalent and trivalent metal cations are evenly mixed in the pristine LDH. Second, the specific surface area increases, and porous structure can be obtained. The metal oxide domains produced during thermal treatment can be connected by the trivalent metals migrated to tetrahedral site [23,24], resulting in intra-particle mesopores [25,26,27]. It is generally known that LDHs prepared by conventional coprecipitation method tend to have specific surface area (S_BET_) around 20–60 m^2^/g [28,29,30], while the corresponding LDO had S_BET_ higher than 100 m^2^/g [27,30].

The specific surface area of LDO could be controlled by adjusting synthetic conditions such as controlling calcination temperatures [31,32,33,34]. Generally, increasing temperature produced high specific surface area. In addition, it was also reported that the physical parameter of starting material (LDH) such as particle size, crystallinity, and aspect ratio affected the S_BET_ and pore size [27,35]. However, the range of control for pore and S_BET_ was fairly narrow in aforementioned research. Dramatic increase in surface area could be achieved by applying specific synthetic techniques. For example, the sol-gel method was used to enhance S_BET_ by forming hierarchically stable and integrated structure among metal oxide particles [13,14,15]. Hernandez et al. reported that Ba_3_Li_2_Ti_8_O_20_ prepared by sol-gel method showed S_BET_ = 66.5 m^2^/g through scaffold structures made of metal oxide particles. Another approach to obtain high specific surface area is to synthesize LDH precursor with high S_BET_ value. When precipitated LDH was washed with aqueous miscible solvent such as acetone and methanol, water moiety in interlayer space and surface of LDH was removed resulting in the exfoliation of nanosheets. This method produced LDH precursors with S_BET_ = 365 m^2^/g [36,37,38].

In order to control pore size more precisely, the sacrificial template method could be applied. In fact, this method is generally adopted in the synthesis of porous inorganic materials. Silica particles with homogeneous mesopores such as MCM-41, SBA-15, and SBA-16 were prepared by the assistance of cetyltrimethylammonium bromide, P123, and F127, respectively. Recently, biogenic materials are utilized as sacrificial template due to the small size, homogeneity, periodic structures, and abundance in nature. Porous tricalcium phosphate was prepared by utilizing protein as templates [39,40]. Similarly, CeO_2_ catalyst or nanosized NiO could be synthesized through sacrificial template of albumin [41,42]. Deoxyribonucleic acid was utilized to prepare Co_9_S_8_ nanoparticles with mesoporous internal structure [43], or to synthesize porous transition metal oxide [44]. It was also reported that porous alumina was obtained by collagen. Besides, carbon nitride catalyst such as g-C_3_N_4_ was prepared with melamine template. [45,46]. The above-mentioned strategy of sacrificial template could be applied to the synthesis of porous LDO starting from LDH. Homogeneously incorporated sacrificial template between LDH particles can be decomposed during calcination producing homogeneously distributed pores in the network of LDH particles. For instance, micelle-forming surfactant such as dodecyl sulfonate was utilized to induce discrete mesopores ~5 nm [47]. Pluronic polymers such as F127 and P123 were utilized to produce mesopores and high specific surface area [10,48,49]. However, to the best of our knowledge, there have been few reports on bio-templating method to produce porous LDO from LDH. Interfacial interaction between proteins and LDH have attracted interests in terms of colloidal stability and biological inertness. LDH particles coated with albumin are known to show high dispersibility and biocompatibility physiological condition both in vitro and in vivo [50,51,52]. It is straightforward that this protein could attach on the surface of LDH to separate individual particles. However, it is challenging to prevent LDH particles from aggregation during removal of protein template by calcination. In order to overcome the drawback, it is highly required to homogenously incorporate protein into the inter-particle space of LDH during precipitation reaction and to remove templates preserving inter-particle networks.

In this study, we designed a synthetic procedure to take maximum advantage of albumin as sacrificial template and to address the above-mentioned obstacles. First, albumin was incorporated into LDH during conventional coprecipitation synthesis. The action of albumin as template was investigated by comparative characterization before and after calcination. The LDO material was again utilized as a precursor for LDH through reconstruction route in which hydration and anion incorporation occurred simultaneously to recover original LDH phase [53]. The albumin template was again incorporated into LDH during reconstruction, and combusted by thermal treatment again. The reaction condition of reconstruction was different from that of coprecipitation in terms of pH, ionic strength, crystal growth mechanism, etc.; therefore, the physicochemical status of albumin in LDH at each synthesis step would be different accordingly. Two types of LDHs and LDOs were thoroughly characterized to suggest not only the possibility of albumin as biological template but also the optimum synthetic condition of albumin-based porous LDO.

## 2. Materials and Methods

### 2.1. Materials

Magnesium nitrate hexahydrate (Mg(NO_3_)_2_·6H_2_O), aluminum nitrate nonahydrate (Al(NO_3_)_3_·9H_2_O), albumin from human serum were purchased from Sigma-Aldrich, Inc. (St. Louis, MO, USA). Sodium hydroxide (NaOH) and sodium bicarbonate (NaHCO_3_) were obtained from Daejung Chemicals & Metal Co., Ltd. (Siheung, Korea).

### 2.2. Preparation of LDHs and LDOs: AH-1, AO-1, AH-2 and AO-2

Albumin-templated LDH (AH-1) was first synthesized following conventional coprecipitation method [54]. Typically, 30.8 g of Mg(NO_3_)_2_·6H_2_O (0.12 mol) and 22.5 g of Al(NO_3_)_3_·9H_2_O (0.060 mol) were dissolved in 250 mL of deionized (DI) water. Alkaline solution containing 38.4 g of NaOH (0.96 mol) and 80.6 g of NaHCO_3_ (0.96 mol) was prepared with 500 mL of DI water. Both solutions were injected simultaneously into 250 mL of albumin solution (0.5 g/L). The injection rate of metal and alkaline solution was carefully adjusted in order to control the pH of total reaction system constant at ~9.5. After 24 h of reaction in room temperature, the suspension was centrifuged at 7000 rpm for 5 min to obtain LDH precipitate, which was thoroughly washed with DI water to remove unreacted ion and salt. The white AH-1 powder was obtained by lyophilization. The calcined LDH (AO-1) was obtained by thermally treating AH-1 at 500 °C for 10 h with 0.8 °C/min of heating rate in a muffle furnace.

For the albumin-templated LDH through reconstruction, 13.0 g of NaHCO_3_ (0.325 mol) was dissolved in 1.5 L of albumin solution (0.5 g/L) to which 6.64 g of AO-1 powder was suspended. After 1-day reaction at room temperature, the precipitate was separated by centrifugation and washed with DI water. The lyophilized product was designated AH-2. Calcination of AH-2 at 500 °C for 10 h with 0.8 °C/min of heating rate resulted in AO-2.

### 2.3. Characterization

The pH-dependent zeta potential of albumin was measured with ELSZ-1000 (Otuska electronics, Osaka, Japan) in plastic capillary cells rinsed with DI water between each measurement. Human serum albumin was dissolved in DI water (0.1 mg/mL) and the pH was controlled by adding appropriate amount of HCl solution (0.1 M) or NaOH solution (0.01 M). All the samples were equilibrated at 25 °C in the instrument before measurement. The electrophoretic mobility of each sample was measured three times and averaged.

The crystal structure was analyzed by powder X-ray diffraction (XRD, D2 Phaser, Bruker AXS GmbH, Karlsruhe, Germany) with Ni-filtered CuKα X-ray (λ = 1.5406 Å). Diffractograms were obtained with time-step increments of 0.02° and step size of 0.2 s. Cell parameters of samples were calculated by Unit Cell (©Tim Holland and Simon Redfern, Cambridge, England). The crystallite size was calculated from Scherrer’s equation [55], based on the peaks at certain direction.

τ = (0.9 λ)/(Bcosθ)

τ: crystallite size (Å), λ: X-ray wavelength (1.5406 Å), B: Full width at half-maximum (FWHM) and θ: Bragg angle.

The particle size and shape were observed by scanning electron microscopy (SEM, FEI QUANTA 250 FEG, Hillsboro, OR, USA) at 30 kV acceleration voltage. Powdered sample was spread directly on the sticky carbon tape, and loosely bound moiety was gently blown away by hand-blower. Then, the surface of sample was coated with Pt/Pd by plasma sputtering for 50 s.

Solid-state ^27^Al magic angle spinning nuclear magnetic resonance (^27^Al MAS NMR) spectra were measured by 400 MHz solid state NMR spectrometry (AVANCE III HD, Bruker, Karlsruhe, Germany) with Larmor frequency 194.29 MHz at the KBSI Western Seoul Center, Korea. The spectra were acquired utilizing 1 pulse (1.2 μ, 45° deg), with a spinning rate of 14 kHz and 1024 scan number. The repetition time of measurement was 2 s. Chemical shifts are quoted in ppm from AlCl_3_(aq).

N_2_ adsorption-desorption isotherms were analyzed through Belsorp-mini (Microtac BEL, Inc., Osaka, Japan) to investigate specific surface area and pore size distribution. All the samples were pre-treated at 100 °C under vacuum condition for 12 h for degassing. The S_BET_ was calculated based on Brunauer–Emmett–Teller (BET) theory. The pore information was estimated through Barret–Joyner–Halenda (BJH) model.

## 3. Results and Discussions

**Figure 1:**
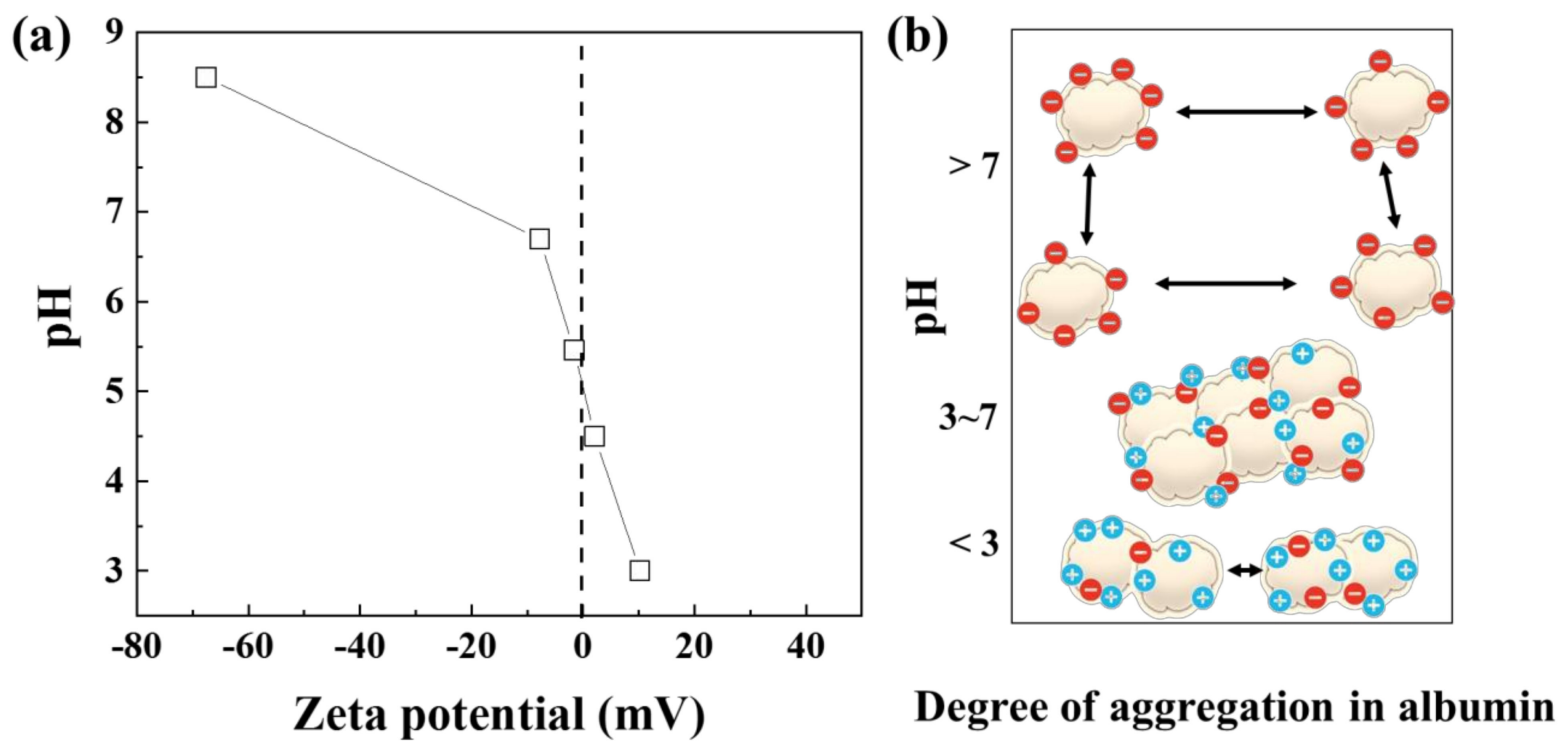
(**a**) Effect of pH on zeta potential of albumin and (**b**) schematic diagram describing the degree of aggregation depending on pH.

**Scheme 1:**
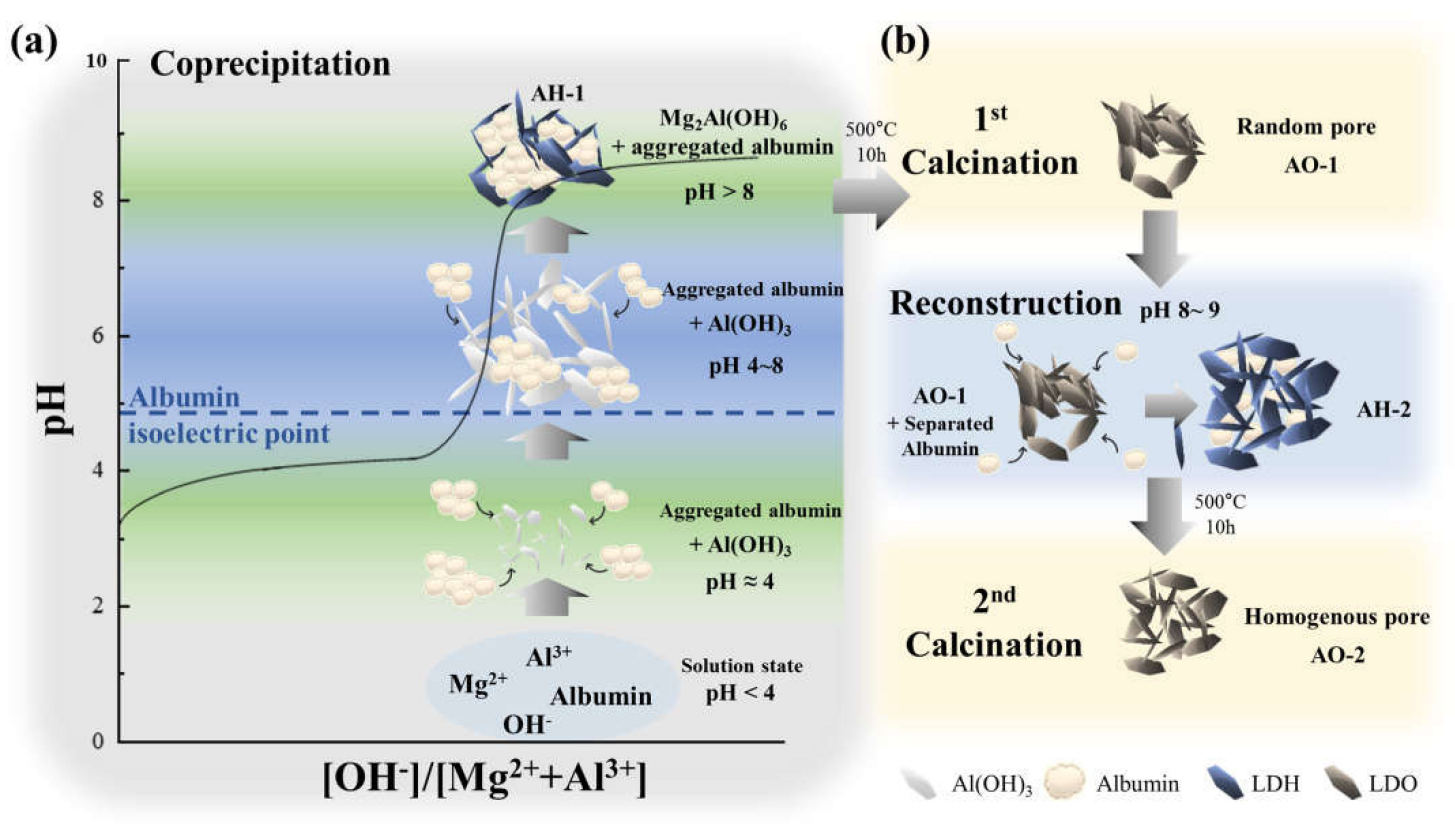
Proposed mechanism of albumin-template during the formation of layered double hydroxide (LDH) or layered double oxide (LDO) under (**a**) coprecipitation and (**b**) reconstruction reaction.

The surface charge of albumin was highly dependent on pH of solution, showing isoelectric point 5.0 as previously reported [56]. Below pH 3 and above pH 7, the zeta potential of albumin was slightly positive and highly negative, respectively, implying the electrostatic repulsion among molecules (Figure 1a). Additionally, we could expect thorough separation of single protein at basic condition compared with neutral or acidic media (Figure 1b pH > 7). In the pH range 3–7, the absolute zeta potential value is below 20 mV and thus albumin molecules tend to form aggregates. This discrepancy of surface charge in albumin at different pH was confirmed by analyzing DLS (Figure S1). Albumin showed strong aggregation (hydrodynamic radius R_H_ = 123.2 nm) at around isoelectric point, while almost single protein was separated (RH < 10 nm) above the isoelectric point. Aggregation of albumin due to neutral charge caused larger particle size than the negative- or positive-charged state. The pH-dependent aggregation of albumin is an important factor when it is utilized as sacrificial template in LDH synthesis, as a set of reactions occur along pH in coprecipitation [57]. First, metal cations are well solubilized under acidic condition (pH < 4); second, upon addition of basic solution, trivalent cations form hydroxide (pH~4); the divalent metals are incorporated in trivalent metal hydroxide lattice at pH 8–9 to form mixed metal hydroxide, i.e., LDH (Scheme 1a). It is expected that the aggregated albumin lumps and Al(OH)_3_ crystallites are mixed together in the pH range 4–8, and the mixed status would continue until LDH lattice forms at high pH. The heterogeneous aggregates of albumin during the coprecipitation of LDH can hinder their action as homogenous template (Scheme 1a). As a result of albumin aggregation, heterogeneous inter-particle pore would form after calcination (AO-1 in Scheme 1b). On the other hand, in the reconstruction route, the pH is maintained constantly between 8 and 9 where albumin molecules can be separated from each other due to highly negative surface charge (Figure 1a). During reconstruction of AO-1 to AH-2, the protein moiety would homogeneously attach to the particle surface to play a role as a template for homogenous pores (AH-2 in Scheme 1b).

**Figure 2:**
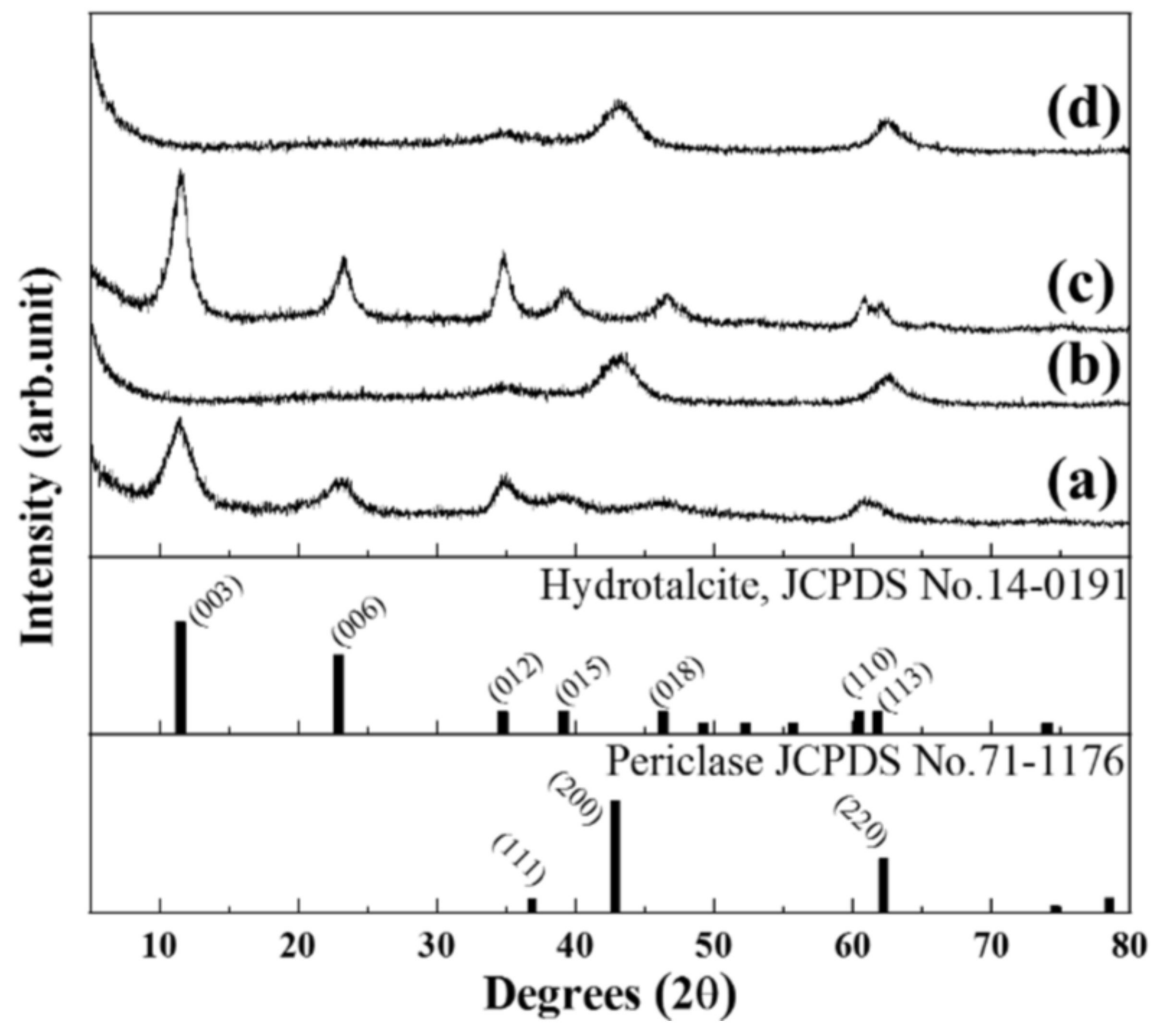
XRD patterns of (**a**) AH-1, (**b**) AO-1, (**c**) AH-2, and (**d**) AO-2. Joint Committee of Powder Diffraction Standards (JCPDS) No. 14-0191 and 71-1176 stand for hydrotalcite and periclase phases, respectively. Hydrotalcite is a mineral name of naturally found Mg_3_Al(OH)_8_(CO_3_)_0.5_-LDH. Periclase is a mineral name of MgO, which is the major component of calcined MgAl-LDH.

The crystal structure of albumin-templated LDH and the LDO was investigated by X-ray diffraction patterns. As shown in Figure 2a,c, both albumin-templated LDHs showed typical hydrotalcite crystal structure (JCPDS No. 14-0191), with diffraction peaks at 2θ of 11.48°, 23.23°, 34.76°, 39.25°, 46.65°, 60.83°, and 62.04° corresponding to (003), (006), (012), (015), (018), (110), and (113) planes, respectively. The d-spacing of both albumin-templated LDHs were the same as 7.70 Å, which indicated existence of carbonated anion in interlayer of LDH. This clearly showed that the albumin moiety did not enter the gallery space of LDH layer. As the zeta potential value of AH-1 (Figure S2) was less positive than that of conventional LDH, we expected albumin moiety adsorbed on the surface of LDH particles. Lattice parameters of both LDHs were same (a = 3.04 Å, c = 23.2 Å), suggesting that the different synthesis route (coprecipitation for AH-1 and reconstruction for AH-2) did not significantly affect the crystal formation of LDH. However, we could observe that the X-ray diffraction intensity of AH-1 was lower than that of AH-2 at all the diffraction peaks. Scherrer’s equation quantitatively showed the larger crystallite size in the reconstructed one. Crystallite sizes along (00l) direction slightly increased from 3.50 nm (AH-1) to 5.33 nm (AH-2); while the crystallite size along (110) direction dramatically increased from 3.84 nm (AH-1) to 12.29 nm (AH-2). The results clearly showed that the crystal growth of LDH was more facilitated under reconstruction than coprecipitation. As described in Figure 1, albumin underwent serious pH change during coprecipitation of LDH. It is considered that the albumin aggregates prevented LDH from effective crystal growth. On the other hand, the reconstruction reaction occurs at relatively basic pH, at which albumin proteins could exist in individual molecule. Albumin moiety during AH-2 synthesis could be homogenously adsorbed on the LDH surface, not seriously hindering crystal growth. The crystal growth in AH-2 was more accelerated *ab*-plane direction than *c*-axis, as the surface adsorbed albumin could hinder the effective layer stacking in LDH formation.

Calcined LDHs, AO-1 and AO-2, were determined to have periclase (MgO) phase (JCPDS No.71-1176), exhibiting diffraction peaks at 2θ of 34.53°, 43.12°, and 62.52° for (111), (200), and (220), respectively (Figure 2b,d). The lattice parameter was same for both LDOs (*a* = 4.19 Å). Furthermore, the crystallite size of AO-1 and AO-2 were determined 3.52 nm and 3.75 nm, respectively, along (220) direction, showing that there was no significant effect of the LDH’s crystallinity. This finding is similar to our previous report [27], where we suggested that the crystallite size of LDO was affected by neither the particle size nor crystallinity of pristine LDH.

**Figure 3:**
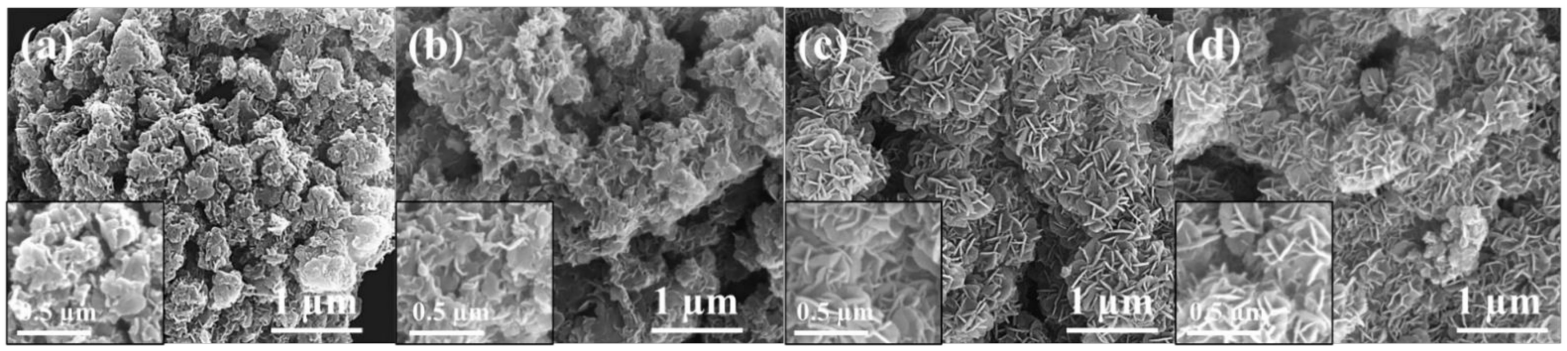
SEM images of (**a**)AH-1, (**b**) AO-1, (**c**) AH-2, (**d**) AO-2.

The size, shape, and arrangement of particles were investigated through scanning electron microscopy. As displayed in Figure 3a, AH-1 had irregularly shaped particles of which the size was less than 100 nm. Compared with the other three samples, AH-1 did not show clear grain boundaries, which might be attributed to the large lumps of organic moiety [53,58,59] like proteins. After calcination (AO-1), particle morphology of ruffled layers appeared along with the combustion of albumin-template (Figure 3b). Although it was not easy to determine the particle dimension precisely, the lateral size and thickness of a particle was approximately 50 nm and 13 nm, respectively, suggesting anisotropic shape. The reconstructed LDH with albumin template (AH-2) began to clearly show sand-rose structure, which was a typical feature of reconstructed LDH (Figure 3c) [35]. The lateral particle size increased up to ~135 nm, which was parallel to the preferential crystal growth along *ab*-plane as shown in the XRD data (Figure 2). The sand-rose arrangement was attributed to the edge to face interaction among plate-like LDH particles (Figure 3c) [35]. The grain boundary of AH-2 was clearer than that of AH-1 in SEM images, and thus it was expected that protein moiety was attached on the surface of AH-2 without formation of significant aggregates. The size, shape, and arrangement of AO-2 particles were fairly comparable with those of AH-2 (Figure 3d), suggesting that the protein decomposition did not seriously deteriorate LDH particle. As previously reported, phase transformation from LDH to LDO tended to occur in intracrystalline manner while the overall particle morphology was preserved [27,35].

The AH-2 and AO-2 apparently exhibited inter-particle spaces, enabling us to expect their higher specific surface area than the coprecipitated ones (AH-1 and AO2). In addition to the inter-particle pores, the AO-2 would have intra-particle mesopores, which can be formed by the thermal decomposition of albumin. As the distribution of albumin in AH-2 was more homogeneous than that in AH-1, the mesopore distribution of AO-2 would be clear compared with AO-1.

**Figure 4:**
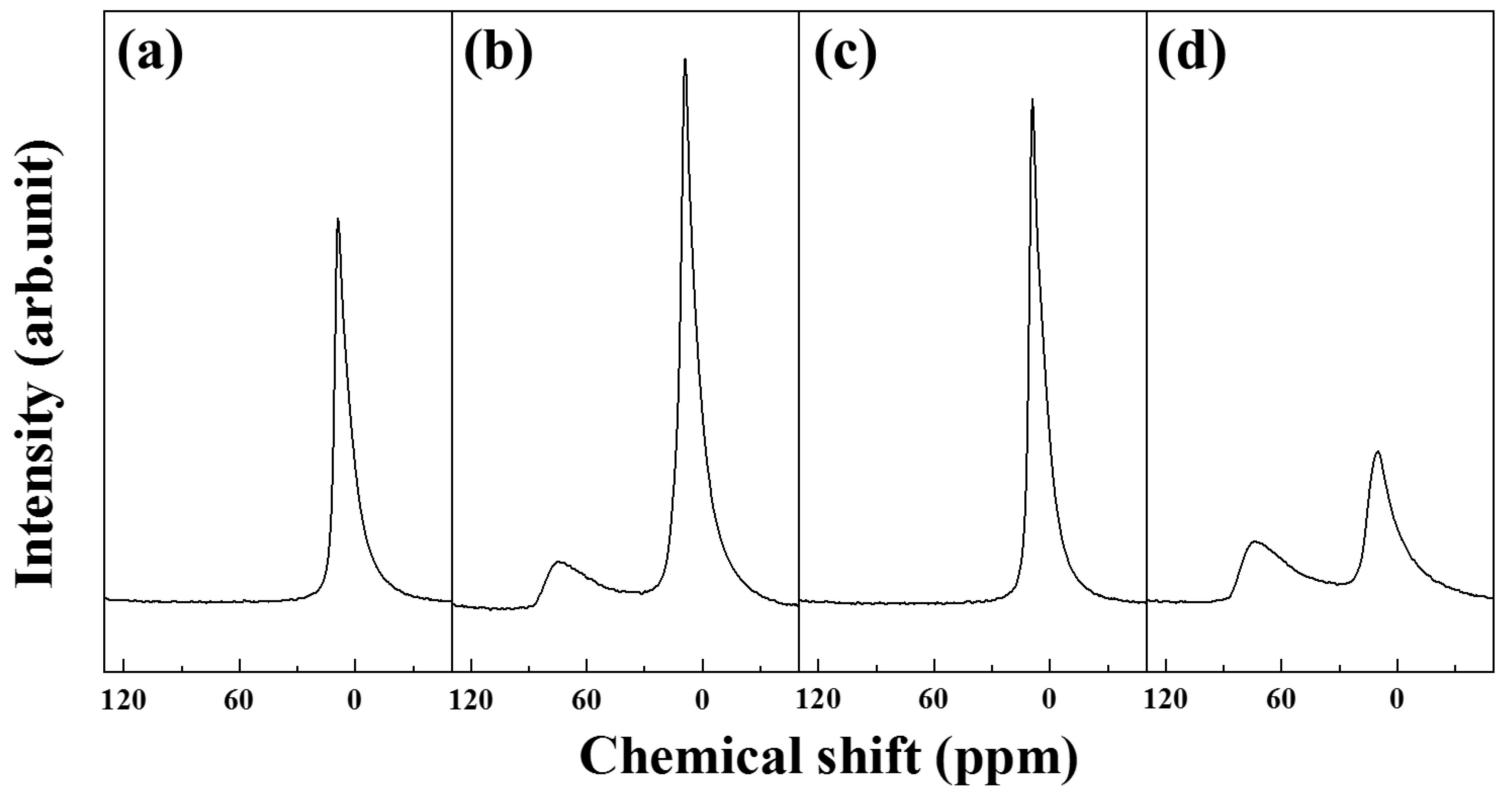
Solid state 27Al MAS-NMR spectra of (**a**) AH-1, (**b**) AO-1, (**c**) AH-2, and (**d**) AO-2. Peaks at around 10 ppm and 75 ppm were assigned to Al3+ (O_h_) and Al3+ (T_d_), respectively.

Chemical environment around Al^3+^ in LDHs and LDOs was examined with the ^27^Al solid state MAS NMR spectroscopy, as the local disorder was reported to have strong relationship with pore development. The NMR spectrum of AH-1 (Figure 4a) showed a sharp signal at 9.20 ppm, which corresponded to 6-coordination Al(OH)_6_ in LDH [35]. Upon calcination, a broad peak at around 74.3 ppm appeared, indicating the formation of 4-coor-dinate Al^3+^ environment (Figure 4b). It is known that the Al^3+^ in the octahedral (O_h_) site of LDH partially migrates to tetrahedral site (T_d_) to interstratify MgO domains [22,23,27,35]. It is noticeable that the peak area ratio T_d_/O_h_ of 0.145 (Table 1) in AO-1 was lower than the previously reported values ranging 0.59–1.32 [27,35], indicating that local disorder around Al^3+^ was less severe than usual. Although it is not clear why the O_h_ to T_d_ migration of Al^3+^ was limited in AO-1, we could at least expect that the large aggregates of albumin among LDH particles would negatively influence the interstratified arrangement of MgO domains by Al^3+^ (T_d_), possibly resulting in less benefit in pore development. The interconnection of MgO domains is important in developing porous structure in LDO, as the inter-domain space could act as pores. Reconstruction reaction on AO-1 recovered the original 6-coordinate of Al^3+^, which was evidence by the strong signal at 8.92 ppm (Figure 4c). There was no significant peak corresponding to 4-coordinate Al^3+^, implying perfect recovery of original coordinate structure. The peak for O_h_ in AH-2 was almost identical to that of AH-1 in terms of position, shape, and intensity, suggesting that the local disorder of both samples was similar. On the other hand, the spectrum of AO-2 exhibited both significant reduction in O_h_ peak intensity as well as prominent appearance of T_d_ peak (Figure 4d). The ratio of peak area T_d_/O_h_ in AO-2 was 0.589 (Table 1), which was comparable with the previous reports [27,35]. Different from AH-1, the albumin molecules in AH-2 are thought to exist fairly homogeneously at the molecular level. As the local structural change was not seriously interfered, AO-2 would have well-developed interstratified MgO domains connected by Al (T_d_). It could be therefore concluded that the AO-2 could take advantage of high specific surface area with homogeneous porosity.

**Figure 5:**
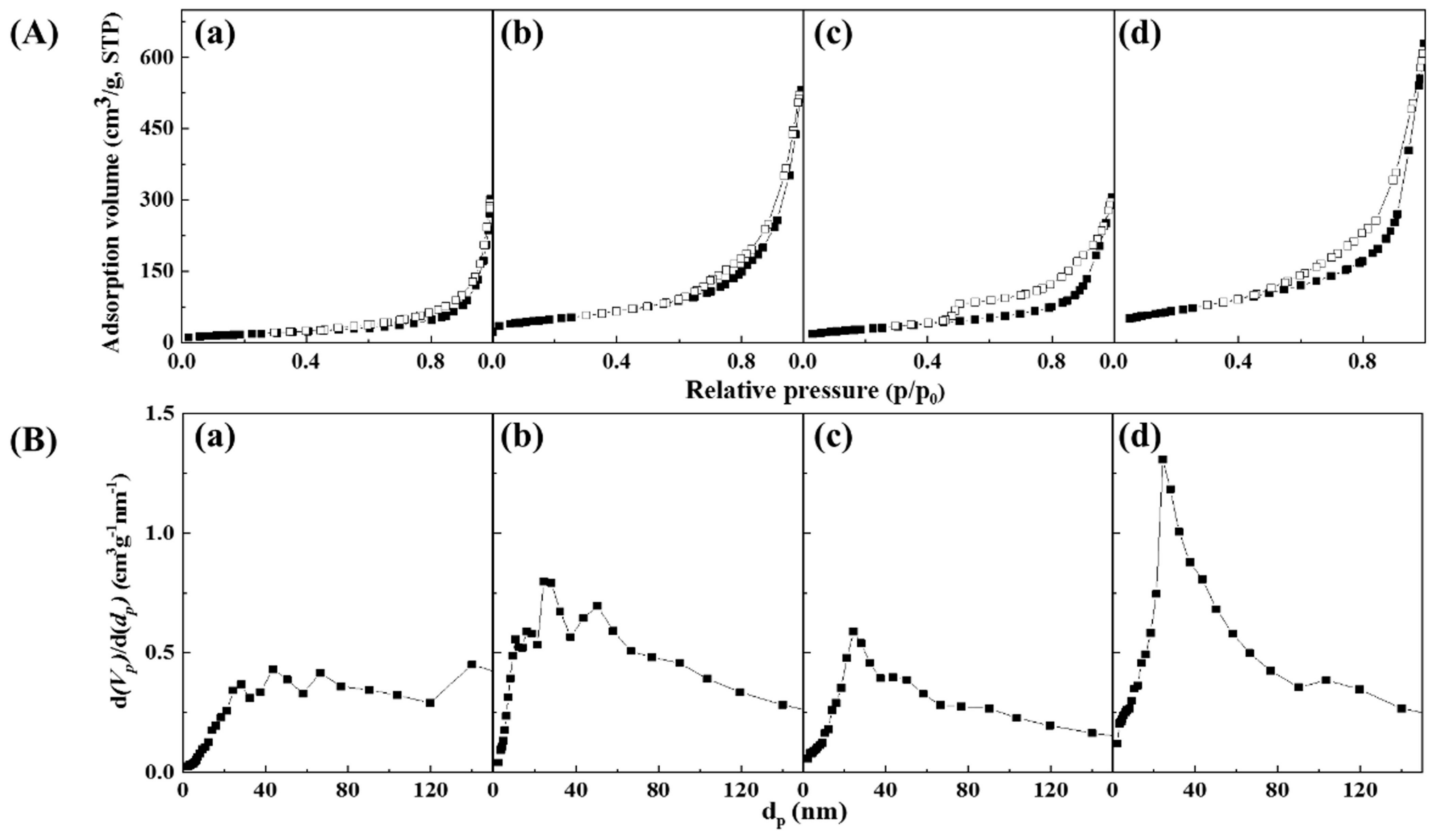
(**A**) N2 adsorption-desorption isotherms and corresponding (**B**) BJH pore size distribution plots for (**a**) AH-1, (**b**) AO-1, (**c**) AH-2, and (**d**) AO-2.

In order to evaluate the specific surface area and porous structure of LDHs and LDOs prepared by two different synthesis methods, N_2_ adsorption-desorption isotherms were analyzed (Figure 5A). The adsorption type of LDHs and LDOs can be assigned to either type III or II, respectively (Table 1), according to the International Union of Pure and Applied Chemistry (IUPAC, Research Triangle Park, NC, USA) classification [60]. Type III is a characteristic of non-porous materials with low adsorbent-adsorbate interaction, while type II refers to polymolecular adsorption on macroporous material (Figure 5A(a,c)). LDH itself only provides its particle surface as adsorption site, and thus it is considered as non-porous material. On the other hand, LDO could have intra-particle pore due to the dehydroxylation or decomposition of albumin-template, resulting in type II adsorption (Figure 5A(b,d)). This was also confirmed by the higher specific surface area and pore volume of LDO than LDH (Table 1). The S_BET_ value of AO-1 and AO-2 was 2.7 and 2.1 times larger than their LDH form. Pore volume also showed similar pattern: AO-1 and AO-2 had 1.9- and 2-times larger pore volume than LDH counterparts.

Beside the difference in porosity between LDH and LDO, we could also observe distinctive characters of reconstruction samples (AH-2 and AO-2) compared with coprecipitation ones (AH-1 and AO-1). First of all, AH-2 and AO-2 contained clear hysteresis in Figure 5A(c,d), which were assigned to H4 and H2, respectively, based on IUPAC classification (Table 1). Both hysteresis types are often reported in LDHs and LDOs, which were especially designed to have high specific surface area and controlled pore [61,62]. Hysteresis types H4 and H2 indicate slit-like pore and ink-bottle pore, respectively [63,64,65]. AH-1 and AO-1, in which we expect strong aggregation of albumin, did not show certain types of hysteresis due to the following reason. Large lumps of albumin in AH-1 blocked the inter-particle space of LDH particles, preventing development of shaped pores. In AO-1, despite removal of protein template, the space that was filled with large albumin aggregate could be not maintained, hindering formation of well-developed mesopores. On the other hand, evolution of H4 hysteresis in AH-2 [35] could be explained by the predominant crystal growth of LDH along crystallographic *ab*-plane upon reconstruction. Highly anisotropic particles of reconstructed LDH were randomly oriented giving rise to slit-like pore. The ink-bottle pore of AO-2 might be attributed to the thermal decomposition of homogeneously dispersed albumin in addition to the pre-existing slit-like pore. The BJH pore size distribution of AO-2 was fairly narrow and showed that major pore size was 14.9 nm, which was corresponding to mesopore in Figure 5B(d). Compared with Figure 5(c), the evolution of mesopore in AO-2 implied that the albumin moiety efficiently acted as the template for mesopores. On the other hand, the coprecipitated LDH (AH-1) was mixed with aggregated albumin in Figure 5B(a), and thus the calcination (AO-1) did not significantly induce mesopores in Figure 5B(b).

## 4. Conclusions

We successfully utilized a biogenic molecule, albumin, as a sacrificial template to develop mesopores in LDO by calcining albumin-containing LDH. Although there have been research studies on the surface adsorption of albumin on LDH surface for colloidal stability, it was fairly challenging to maintain inter-particle network during removal of protein by thermal treatment. In order to address the problem, it is required to incorporate protein moiety into the LDH particle system in homogeneous manner. At the same time, it was necessary to remove proteins while preserving interparticle spaces. It was found that the pH condition was an important factor to control the aggregation status of albumin and thus the synthetic procedures both direct coprecipitation and reconstruction were applied for comparison. Through zeta-potential, we confirmed that albumin showed pH dependent intermolecular electrostatic interaction. Albumin formed aggregates during coprecipitation process, while it can be homogenously distributed on LDH surface during reconstruction process. The XRD patterns indicated that albumin aggregation could prevent crystal growth of LDH in coprecipitation, whereas the crystal growth of LDH along lateral direction was accelerated by the surface adsorbed albumin in reconstruction condition. Both LDOs showed similar crystal structure and crystallite size but different morphologies. According to the SEM images, we verified the lateral particle size increased in reconstructed LDH. Furthermore, the AH-2 and AO-2 showed uniform inter-particle cavity, and thus high specific surface area was expected. The ^27^Al solid state MAS NMR spectra revealed local disorder, one of the driving forces to produce pores in LDO, was restricted in AO-1, possibly due to the negative effect of albumin aggregates. On the other hand, AO-2 showed facilitated local disorder around Al^3+^, resulting in the production of mesopores. The N_2_ adsorption-desorption isotherms verified that the specific surface area of LDO was larger than LDH precursor, suggesting the general action of calcination to produce pore in LDO. In addition, BJH pore size distribution clearly indicated narrower distribution of mesopore in AO-2 than in AO-1. This might be attributed to the combustion of homogeneously dispersed albumin in addition to the dehydroxylation of LDH. It is therefore concluded that the albumin could be utilized as sacrificial template to produce LDO with mesopore and that the synthetic condition of LDH should be controlled to obtain pore structure with narrow size distribution.

## Figures and Tables

**Table 1:** Physicochemical parameters obtained from solid state ^27^Al MAS-NMR spectra and N_2_ adsorption-desorption isotherms.

Sample	Al-NMR			N_2_ Adsorption-Desorption Isotherm			
	Peak Position (ppm)		Peak Area Ratio (T_d_/O_h_)	Adsorption Type	Hysteresis Classification	S_BET_ (m^2^/g)	Pore Volume (cm^3^/g)
	O_h_	T_d_					
AH-1	9.20	-	-	III	-	65.2	0.439
AO-1	9.40	74.3	0.145	II	-	176.6	0.801
AH-2	8.92	-	-	III	H4	107.8	0.463
AO-2	10.1	73.4	0.589	II	H2	224.3	0.899

## Data Availability

The data presented in this study are available on request from the corresponding author. The data are not publicly available due to privacy.

## Supplementary Materials

The following are available online at https://www.mdpi.com/2079-4991/11/3/620/s1, Figure S1: Hydrodynamic radius of albumin in protonation and deprotonation state, Figure S2: Zeta potential of AH-1 and LDH pristine.
