# Supplementary material for: Development of Mesopore Structure of Mixed Metal Oxide through Albumin-Templated Coprecipitation and Reconstruction of Layered Double Hydroxide
Source: Nanomaterials (Basel). 2021 Mar 2;11(3):620. doi: 10.3390/nano11030620 (PMC7999424; doi:10.3390/nano11030620)
Supplement: Supplementary file 1 [file nanomaterials-11-00620-s001.pdf]

Supplementary information

Development of Mesopore Structure of Mixed Metal Oxide Through Albumin-Templated Coprecipitation and Reconstruction of Layered Double Hydroxide

Sang-Yong Jung[1], Bo-Kyung Kim[2], Hyoung-Jun Kim[3,*], Jae-Min Oh[1,*]

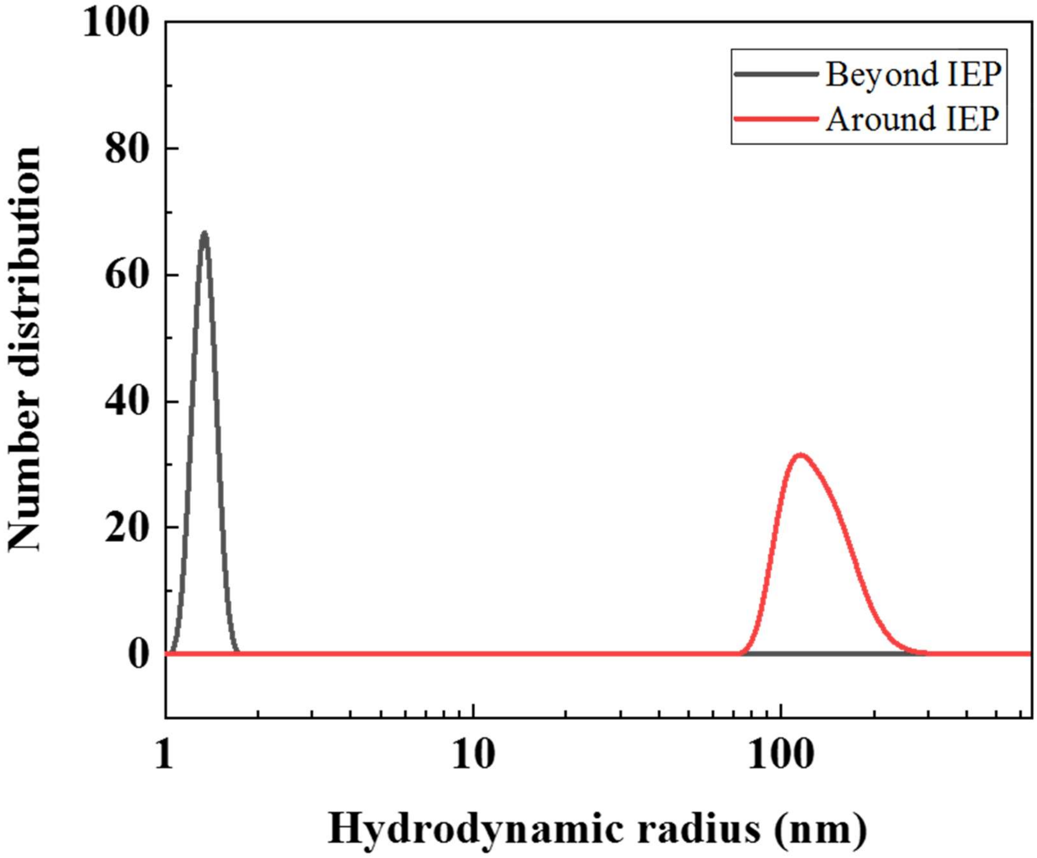

Figure S1. Hydrodynamic radius of albumin in protonation and deprotonation state. IEP indicates isoelectric point of albumin

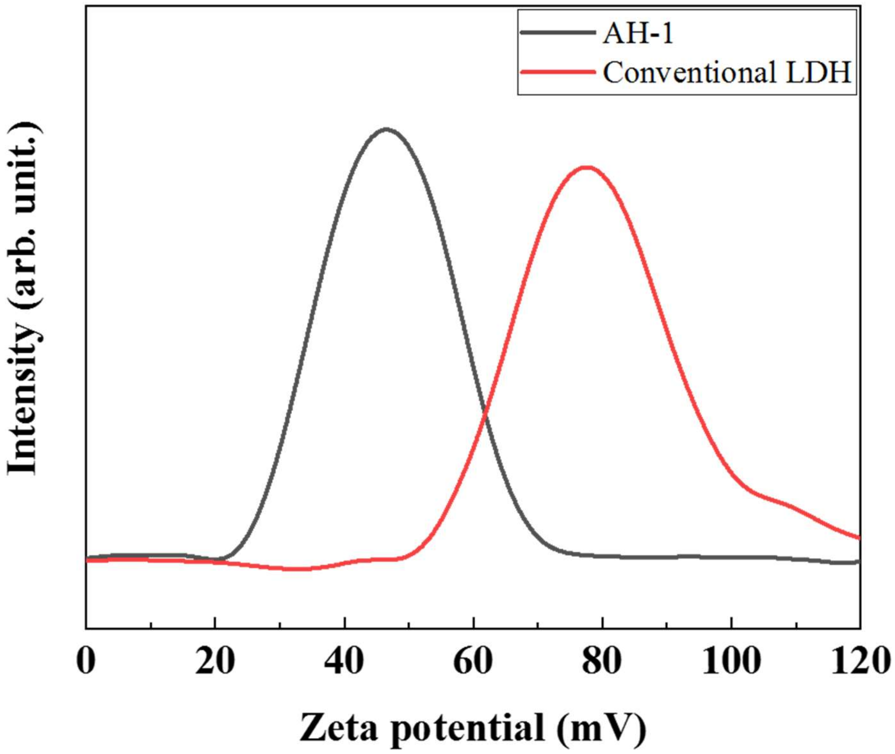

Figure S2. Zeta potential of AH-1 and LDH pristine
